# Scarcity in today´s consumer markets: scoping the research landscape by author keywords

**DOI:** 10.1007/s11301-022-00295-4

**Published:** 2022-09-29

**Authors:** Haoye Sun, Thorsten Teichert

**Affiliations:** Chair of Marketing and Innovation, Hamburg University, Von-Melle-Park 5, 20146 Hamburg, Germany

**Keywords:** Scarcity, Author keywords, Research streams, Consumer behavior, Socio-political, M00, Q00

## Abstract

Scarcity refers to not having enough of what one needs. This phenomenon has shaped individuals´ life since ancient times, nowadays ranging from daily-life scarcity cues in shopping scenarios to the planet’s resources scarcity to meet the world´s consumer demand. Because of this ubiquity of scarcity, the topic has been attracting attention from scholars and practitioners in different areas. Studies regarding scarcity were conducted across disciplines, based on different assumptions, and focused on distinct study subjects. A lack of mainstream about this topic hindered the convergence of core ideas among different schools of thought. In this article, we take an integrative socio-economic perspective to join diverse findings on scarcity affecting consumer markets, identify topic-specific research questions still to be answered, and provide suggestions for future and integrative research opportunities. A systematic review based on author keywords from 855 publications analyzing scarcity affecting business-consumer interactions serves as a database. Exploratory factor analyses based on author keywords identify shared patterns within and linkages across discourses stemming from various disciplines and theories. Results differentiate distinct research foci in the consumer behavior, socio-political, and other disciplinary research realms. A mapping of these research themes identifies the scarcity-related interplay among consumers, producers, and other stakeholders. Findings point out research directions for future studies at both the research realm level and the interdisciplinary level.

**Supplementary Information:**

The online version contains supplementary material available at 10.1007/s11301-022-00295-4.

## Introduction

Scarcity refers to basic limitations in economic transactions resulting from the gap between resource availability and individuals’ needs (Cannon et al. 2019). The notion of “resource” covers a wide range of forms, including commodities, services, profits, energy, water, time, etc. (Goldsmith et al. 2021; Hamilton et al. 2019). It is undeniable that nowadays more individuals in the world have enough material, emotional as well as spiritual resources to satisfy their living needs and development desires. However, even in times of material abundance in the Western hemisphere, scarcity is not an outdated topic for researchers and practitioners. If we look back on our online/offline shopping experience, scarcity appeals such as “limited edition”, “last chance”, and “max 2 products per consumer” will always pop into our minds. Global events also remind us of the universality of scarcity and its impact on consumers and society (Hamilton et al. 2019). Economic development and even recovery after the 2007–2009 global financial crisis are constrained because of the unbalance between consumers´ resource demands and the scarcity of resources supply (Brown et al. 2014). Recently, the Covid-19 pandemic has led to the manifestations of various and unexpected forms of scarcity in consumer markets (Hamilton 2021), such as the scarcity of grocery products (Omar et al. 2021), water scarcity (Boretti 2020), as well as the scarcity of operational resources and capabilities (Shaheen et al. 2022). The scarcity of the above-mentioned resources jointly builds up a society with scarcity and even puts people in a scarcity mindset. All those examples highlight the ubiquity and multiformity of scarcity, making this concept an umbrella framework for socio-economic limitations in today´s consumer markets.

Scarcity causes difficult trade-off situations for consumers, marketers, and policy makers: how to effectively allocate the scarce resources to meet needs (Shi et al. 2020). Scarcity is thereby a fundamental proposition of classic economic theory, which states that economic actors need to treat resources as limited. As a basic economic problem, scarcity attracts researchers to optimize resource planning using mathematical and modeling methods; this provides strategic orientations. Meanwhile, psychologists suggest that scarcity is not a ubiquitous manifestation in reality but a situated phenomenon perceived by economic actors. More specifically, psychological studies show that individuals tend to think and behave differently based on the perceived scarcity of resources (O’Donnell et al. 2021; Shah et al. 2015); this creates business opportunities (Shi et al. 2020). The concept of scarcity has been linked to a wide range of socio-economic research subjects in consumer markets, such as consumer responses (Hamilton et al. 2019), revenue management (Heo et al. 2013), supply chain management (Fleischmann et al. 2020), sustainable consumption (Waris and Hameed 2020), corporate strategy (Zhou et al. 2007), and policy-making (Quesnel et al. 2019). Such an advance in scientific knowledge across a wide variety of disciplines generally motivates the need for evaluation studies assessing interdisciplinary scientific research (Wagner et al. 2011).

The general understanding of scarcity is that the phenomenon contains different dimensions based on different resource conditions (Datta and Mullainathan 2014; Fan et al. 2019). In a recent publication focused on product scarcity, Shi et al. (2020) recognized a variety of scarcity phenomena, including: physical product scarcity, service product scarcity, natural resources scarcity, the scarcity of managerial resources, as well as the scarcity of psychological resources. There is however a lack of studies that capture individuals’ experiences of scarcity across multiple domains (De Sousa et al. (2018). Even review studies so far aggregated empirical findings about single scarcity dimensions, e.g. on the impact of *product scarcity* on consumer responses (Hamilton et al. 2019; Shi et al. 2020), on supply chain management in the era of *natural resource scarcity* (Kalaitzi et al. 2018), as well as on *scarcity of time and mental resources* in the healthy diet field (Jabs and Devine 2006).

We argue a broader perspective is needed to investigate multiple as well as interdisciplinary dimensions of scarcity and their linkages. Focused studies cannot offer a comprehensive framework that captures various aspects of scarcity with all-embracing breadth, let alone can they reveal interconnections of scarcity dimensions and their core concepts across research themes and disciplines. From the perspective of knowledge integration, critical questions can better be answered from a more holistic perspective, and the diffusion of discoveries can be more widely promoted across different research fields (Aboelela et al. 2007). Accordingly, the current paper seeks to link different dimensions of scarcity in consumer markets to provide a systematic review regarding the socio-economics of the umbrella concept of scarcity. Specifically, this paper aims (1) to identify the main existing research streams in the field of socio-economics that address the scarcity of different resource types in consumer markets; (2) to integrate the main findings of these research streams, and categorize them into underlying research realms; (3) to point out future research directions for each research realm; (4) to sketch possibilities of transferring ideas and methods across the identified research realms.

## Methodology

Informetrics provides a set of tools to systematically analyze research and its development over time (Kuntner and Teichert 2016; Wagner et al. 2011). It uses meta-information provided within academic publications to gain insights at an aggregate level of research fields. Typically, co-citation analyses are conducted to map the research landscape of a scientific discipline (Acedo and Casillas 2005; Frerichs and Teichert 2021). This approach bases on the idea that single publications are built upon each other, such that joint references indicate an overlap of underlying research topics. This mode of analysis works well within a scientific discipline, where there are shared protagonists and idea-providers that are jointly been cited by following articles. However, co-citation analysis can fail in mapping an interdisciplinary landscape. Here, the same topic can be addressed from complementary angles, while referring to different protagonists' works. Thus, a lack of co-citations need not imply different topics but may hint at divergent lenses applied in its analysis.

**Fig. 1:**
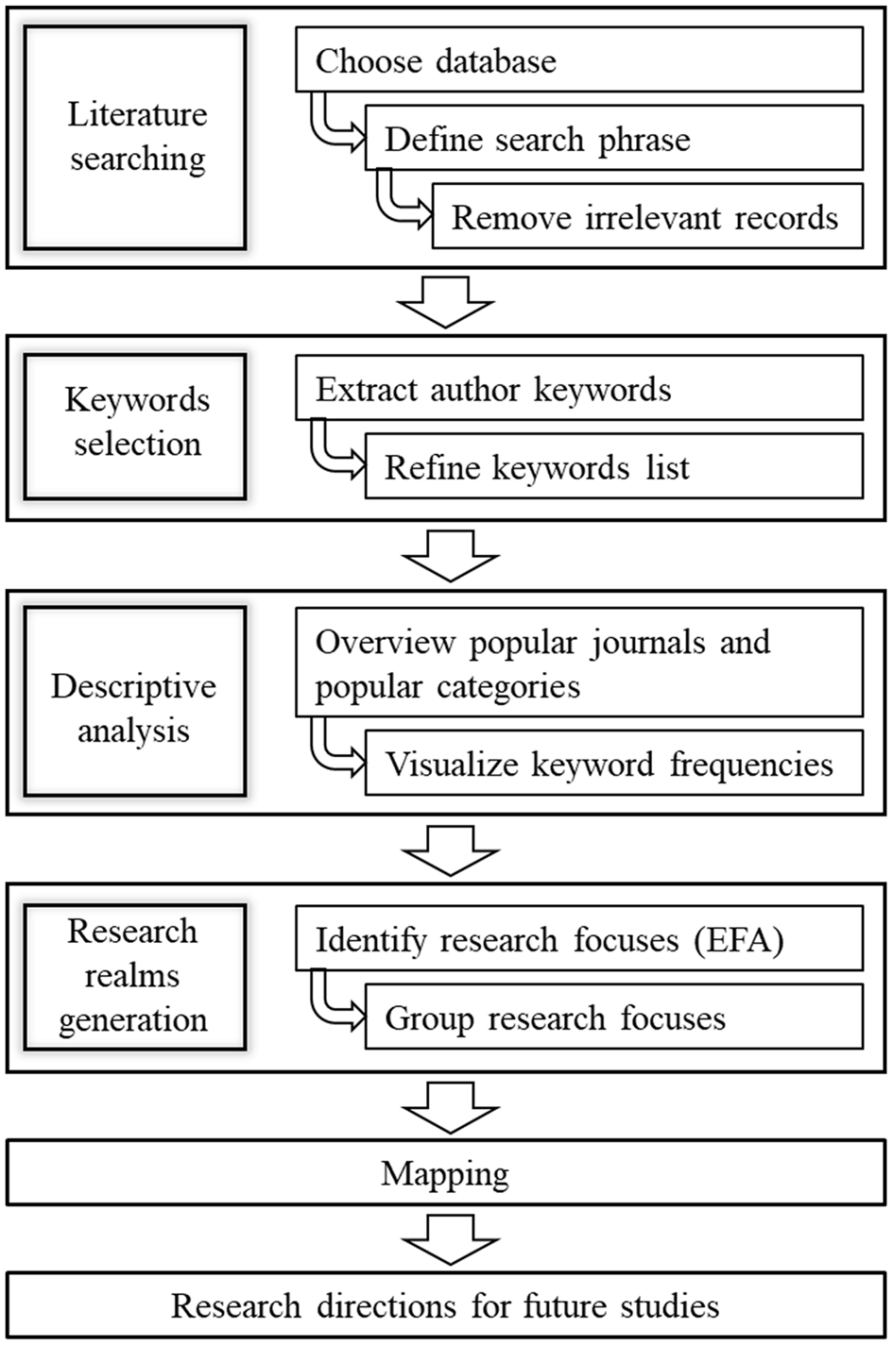
Research framework of the current study

Given the highly heterogeneous and interdisciplinary nature of the scarcity discourse, our paper deviates from common co-citation analysis and instead carries out an informetric analysis based on author keywords. Author keywords refer to the list of topic-specific words hand-picked by the authors to describe the articles´ issues (Lu et al. 2021). These keywords are generally chosen such that they provide general information about the papers´ topics that are been investigated. This holds as author keywords determine the publication success, the paper’s attractiveness to potential readers, and even its dissemination to certain fields. Therefore, authors mostly include informative, most relevant, and refined words with standardized academic expression as keywords (Uddin and Khan 2016). As important entities of meta-data, author keywords play a significant role in bibliographic analysis to clarify scientific knowledge structures, identify subject hotspots, and detect research trends (Lu et al. 2021, 2020). Thus, in this paper, we identify research streams based on author keywords. Figure 1 shows the framework of the paper.

### Data collection

Following standard practice in scientometric research (Chen et al. 2019; Shi et al. 2020), the internationally leading database of Web of Science (WoS) is used to capture the relevant literature. WoS is known to index the influential literature in different fields, thus is regarded as the high-quality database WOS for bibliometric analysis (Shi et al. 2020). Both the Social Sciences Citation Index (SSCI) and the Emerging Sources Citation Index (ESCI) were selected as they jointly represent an especially broad spectrum of the international literature in social sciences (Wörfel 2019). Following the procedure applied by previous articles executing author keywords analyses (Farooq et al. 2018; Keramatfar and Amirkhani 2019), we separated an article search phase from its analysis phase.

In the search phase, a search term was broadly defined to identify relevant articles using based on topics (referred to in either article title, author keywords, or abstract). As stated in the first section, scarcity has been related to various resources in different disciplines. In this paper, we aim to extract the dominant resource types from the existing literature on resource scarcity in each discipline. Thus, we didn’t include the expressions that specify the resource types (e.g., financial dissatisfaction, time pressure, budget contraction, etc.) in our search term. Instead, the noun “scarcity” was used as an elementary search phrase as it constitutes the shared terminological expression used in academic articles (Shi et al. 2020). This search phrase was combined by AND-conditions with additional search phrases relating the topic to issues of consumer markets instead of an engineering or technical angle. For this purpose, we added a second group of search phrases relevant to the perspective of consumers (i.e. consumer* OR customer*). Note that consumers in the service sector of tourism are often labelled by different nouns (Shi et al. 2020; Suri et al. 2007), thus we added tourist-related synonyms “OR tourist* OR traveler* OR traveller* OR visitor*” into the second part of the search term.

To rule out the irrelevant and increase the data quality, the search results were restricted to peer-reviewed publications with the document types as “article”, and language as “English” (Frerichs and Teichert 2021). In particular, irrelevant articles with the expressions of “scarcity of research/ data/ study” were excluded by applying the additional exclusion criteria “NOT scarcity of NEAR/1 research”, “NOT scarcity of NEAR/1 data”, and “NOT scarcity of NEAR/1 stud*”. The refined hit list was exported in August 2021, consisting of 855 articles about scarcity research with the micro-level socio-economic background.

### Data analysis

The data analysis part contains multiple stages. As a first step, descriptive statistics inform about the scope of selected articles and the author keywords used. By doing so, we obtain an initial picture of the interdisciplinary fields and their respective research focuses. Subsequently, a factor analysis is performed to narrow down the overwhelming information derived from hundreds of author keywords to a limited set of underlying research streams. An exploratory factor analysis (EFA) based on author keywords was carried out using the software IBM SPSS Statistics 25. EFA is an established method used to analyze interdependencies among multitudes of variables (i.e., author keywords in our paper) and to derive factors that can capture most of the information of the original variables.

These factors were interpreted as single research streams as follows. Factor loadings (FL) of single keywords inform about the keyword´s usage in a single research stream, i.e. how representative a keyword is for an identified factor (Kuntner & Teichert, 2016). Thus, research streams were characterized by their specific combinations of keywords. To further describe the factors, representative articles were identified based on their usage of these keywords. For each factor, the articles with an especially high absolute and relative number of referenced keywords were identified. A manual inspection of these articles served to describe exemplary works located within the research stream. Joining this bottom-up perspective of referencing individual publications with the top-down perspective of keyword statistics helped us contextualize author keywords and describe their common underlying research streams.

## Results

### Descriptive analysis

The identified 855 articles were published in a huge amount of 500 different journals, distributed across 100 Web of Science Categories. Table 1 illustrates the Top 10 research categories and journals. This confirms a high interdisciplinary, but also reveals a highly dispersed discourse, as few journals contain more than ten publications related to the research topic. Further descriptive analysis across categories indicated that leading journals in environmental sciences (such as Water Research, Journal of Cleaner Production) and business & management (such as Journal of Consumer Psychology, Journal of Business Research) fields have been paying constant attention to scarcity research.

**Table 1:** Top ten popular journals and top ten popular categories

Journals	No. of articles	Web of science categories	No. of articles
Sustainability	29	Business	214
Journal of business research	22	Economics	75
Journal of cleaner production	19	Green & sustainable science & technology	62
Journal of consumer research	18	Hospitality, leisure, sport & tourism	45
Water	11	Environmental sciences	41
Water resources management	11	Management	37
Journal of retailing and consumer services	10	Environmental studies	22
Psychology & marketing	9	Engineering, civil	19
Social behavior and personality	9	Geography	19
International journal of retail & distribution management	8	Public, environmental & occupational health	15

**Fig. 2:**
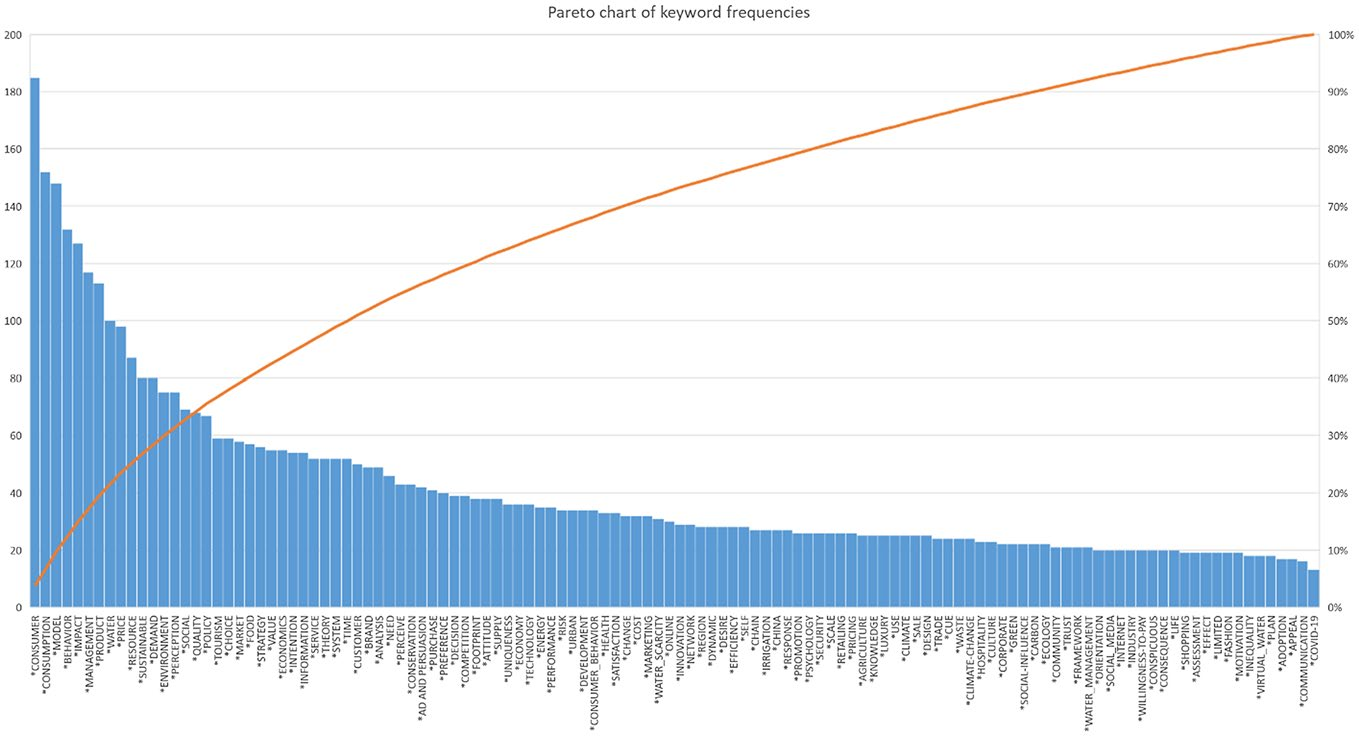
Pareto chart of keyword frequencies

This particular breadth of research works is further illustrated by an analysis of keywords used by the 855 articles. A total of 120 different keywords were identified after word-stemming and cleaning. These keywords also exhibit a high dispersion, as visualized by the low concavity of the Pareto chart in Fig. 2. No single keywords can be identified that are shared across the articles. Even the search-inherent concepts of “consumer” and “consumption” were referenced as author keywords only by a minority of identified articles (22% or 18%), suggesting a more distinct publication focus. Together, this initial inspection of publication metadata shows that standard differentiations by research categories (e.g. environmental sciences versus business & management) are insufficient to characterize the entire socio-economic discourse on consumer market scarcity. Thus, a sophisticated approach of statistical categorization is needed to systematically structure the research field.

### Overview on factor analysis results

A factor analysis is executed to group keywords together based on their co-occurrences. Keywords within one factor are then more likely to co-occur than keywords of different factors. Thus, the keywords assigned to each factor (based on their factor loadings) manifest the key contents of this single research stream. For example, the first factor that captures 18% of the keyword co-occurrence variance is characterized by keywords such as “uniqueness, desire, limited, luxury, social” which indicates purchase-enhancing product scarcity cues. These initial impressions are validated in the following sections by an in-depth analysis of publications belonging to this factor.

Ten factors were extracted by factor analysis to explain 50.03% of the variance in total. Eigenvalue and variance explanation of each factor are presented on the right-hand side of Table 2. Consistent with previous studies in socio-economics (Nooteboom et al. 1997), a cut-off point at the FL value of 0.3 was applied to relate single keywords to their research streams. Table 2 shows the representative keywords under each factor (Table S1 provides the complete list of keywords in each factor). Factor numbers indicate the relative prominence of the identified research stream, measured by factors´ explained variance.

**Table 2:** Overview on research streams (factors) and research realms

#*	Derived title of research stream	Research streams´ keywords	Eigen-value	% of variance
*Consumer behavior research realm: a two-sided view and scarcity*				
F1	Purchase-enhancing product scarcity cues	Uniqueness, desire, limited, luxury, social	20.81	17.34
F3	Dysfunctional effects of product scarcity on consumer behavior	Psychology, response, effect, consequence, decision	5.23	4.36
F5	Scarcity issues in the broader consumption context	Green, time, food, knowledge, health	3.92	3.26
F6	Managing scarcity in the service industries	Customer, satisfaction, hospitality, perception, service	3.13	2.61
*Socio-political research realm: resource scarcity in the water-energy-food security nexus*				
F2	Managing water scarcity	Water, demand, irrigation, policy, water management	12.30	10.25
F4	Footprint as a flow indicator of scarce resources	Footprint, virtual water, assessment, analysis, resource	4.13	3.44
F10	Scarcity and food security	Security, risk, impact, change	2.26	1.89
*Other research realms*				
F7	Competition effects of scarcity	Corporate, orientation, performance, strategy, economy	3.06	2.55
F8	Innovation effects of scarcity	Adoption, trust, internet, technology	2.72	2.27
F9	Optimization models of scarcity	Chain, supply, design, model	2.47	2.06
		Cumulative Variance (%): 50.03		

^*^Factor numbers sorted by descending order of variance explained by factor

To reduce reading complexity, the ten identified research streams are grouped into three overarching realms (subheaders in Table 2). This grouping is based on a robust analysis of between-factor linkages. Hereto, pairwise correlations between keywords belonging to each two different factors are calculated. In Table 3, the negative correlation coefficients indicate that the more author keywords belong to one factor, the less likely they belong to other factors. In other words, the correlation coefficients reflect the possibility of coexistence of articles´ factor belongingness. According to the results shown in Table 3, we grouped the factors with the smallest conflicts (i.e., with the correlation coefficients near zero), resulting in three main research realms (marked with green lines in Table 3). This grouping of research streams (factors) into three overarching research realms is used in the following to arrange the discussion of single research streams. Please note that we nonetheless keep the factor numberings from factor1 to factor10 based on their decreased variance explained.

**Table 3:**
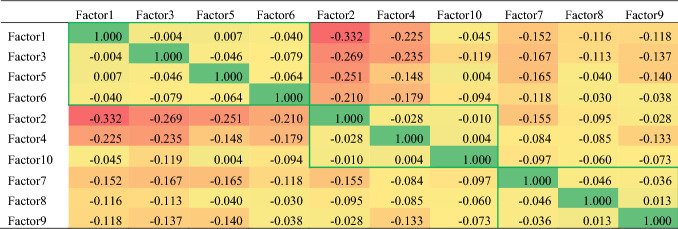
Correlation matrix regarding the distribution of keywords across ten factors (colour Table online)

### Consumer behavior research realm: a two-sided view and scarcity

Studies in consumer behavior often assume that consumers have adequate resources to purchase the products that can meet their consumption goals (Hamilton et al. 2019). However, many consumers experience a scarcity of products/services and/or a scarcity of process resources needed to conduct purchase behaviors (e.g., money and time) (Wang et al. 2021a, b). Intuitively, experiencing insufficiency of what one wants to get is detrimental (Huijsmans et al. 2019), since a failed purchase caused by scarcity restricts consumers’ desire satisfaction (Biraglia et al. 2021). Interestingly, scarcity has also been found to increase consumers’ expectations for scarce products/services, and consequently prompt consumption (Aggarwal et al. 2011; Urbina et al. 2021). These findings seem to contradict each other at first glance, but their inconsistencies can be reconciled by consumers’ attributions of scarcity (Peterson et al. 2020).

In the consumer behavior research realm, we will talk about product scarcity and service scarcity in the retailing and service industries, respectively. Moreover, we will provide an in-depth understanding of the positive and negative effects of scarcity cues on consumer responses, and further explain the underlying mechanism for the two opposite effects.

#### Research stream (F1) on “purchase-enhancing product scarcity cues”

Scarcity cues in this research stream relate to the positive effects of the restricted availability of products or services. Such scarcity cues have been found to positively affect consumer responses, including shaping positive attitudes towards scarce products/brands, purchase intention, and willingness to pay/purchase. Following this line, previous studies confirmed a persuasive effect of scarcity appeals on consumer behaviors (Stock and Balachander 2005). Moreover, scarcity cues motivate non-rational consumer responses, such as luxury experiences seeking and impulsive purchases. That is because scarcity cues can lead to the conclusion of the rarity and uniqueness of products or brands (Chae et al. 2020). Limited-edition products can also address consumers’ desire for uniqueness (Urbina et al. 2021), since consuming unique products is an effective means to express consumers’ uniqueness (Bennett and Kottasz 2013). More importantly, scarcity serves as a differentiator of social class and social status (Bozkurt and Gligor 2019). Accordingly, consumers’ social-related characteristics (e.g., power state, social rejection vs. acceptance, social pressure, wealth) can moderate their reactions to scarcity cues (Bozkurt and Gligor 2019; Kim 2018; Song et al. 2021).

#### Research stream (F3) on “dysfunctional effects of product scarcity on consumer behavior”

This research stream complements the effects analysis of product scarcity on consumer behavior by addressing its possible negative effects. In general, scarcity messages provide urgency information to consumers, which consequently leads to a shorter deliberation and higher purchase amount, higher evaluation, as well as greater satisfaction with the scarce products (Aggarwal et al. 2011). Therefore, one could say scarcity is a “powerful weapon” in marketing. However, scarcity can be accompanied by “darkness” (e.g., negative affect or stress) (Huijsmans et al. 2019), and can even lead to negative perceptions of the scarce products and their sellers (Brannon and Brock 2001). As a spontaneous reaction, scarcity messages can cause negative physiological reactions. For instance, Book et al. (2001) observed the connection between scarcity promotions and testosterone levels, which indicates consumers’ aggressive reactions (Kristofferson et al. 2017).

Scarcity information may also cause a negative image of scarce products or their sellers. When consumers attribute the products’ scarcity information to “demand”, they tend to perceive the products to be unique (Urbina et al. 2021) and of better quality (Parker and Lehmann 2011). However, scarcity messages can also be interpreted as supply-side problems (e.g., stock-out), that evoke negative consequences, for example, lower ratings of the sellers, choice shift, as well as cancelation of purchases (Anderson et al. 2006; Sloot et al. 2005).

In summary, the framing of a product-related scarcity message can evoke completely different psychological processing and behavioral responses. Recent empirical studies provide interesting insights in this regard. Compared with supply-driven scarcity messages, such as “out-of-stock” and “unavailable”, demand-driven scarcity messages (e.g., “sold out”) lead to few negative responses to products and sellers (Kim and Lennon 2011; Peterson et al. 2020). Nevertheless, when consumers attribute scarcity as the result of accidental or non-market forces, neither positive nor negative effects on consumers’ responses are observed (Parker and Lehmann 2011).

#### Research stream (F5) on “scarcity issues in the (broader) consumption context”

In the previous sections, we summarized findings regarding how product scarcity influences consumers’ responses (i.e., attitudes, intentions, and behavior). Another research stream investigates consumers’ responses when facing scarcity in the broader consumption context. Two distinct themes were addressed under this umbrella, time and resource scarcity. A long tradition of research investigated causal relationships between time scarcity and impulsive and dysfunctional consumer behavior. Specifically, time pressure was found to trigger consumers’ impulsiveness, which increases their likelihood of eating ready-to-eat food without thinking about the nutritional ingredient and the following health consequences (Celnik et al. 2012; Machín et al. 2018; Sarmugam and Worsley 2015). More recently, the scarcity of resources (e.g., water, fuel) attracted more attention from researchers. A series of studies tried to shed light on consumers’ purchasing intention/behavior of sustainable products. In these studies, ecological knowledge and awareness of scarcity are found to be the main drivers of green consumption (Waris and Hameed 2020).

#### Research stream (F6) on “managing scarcity in the service industries”

Previous research streams have already covered different dimensions of scarcity based on different resource conditions in retailing. The research stream identified by factor 6 discusses the concept of scarcity in the service industry. Research addresses both positive effects of scarcity on consumer perceptions as well as negative supply-side scarcity effects.

Similar to retailing context, scarcity information may also act as a cue driving consumption in the service industry. The scarcity of specific service offerings, which is regarded as the manifestation of rarity, is confirmed to have a positive impact on customer satisfaction (Moulard et al. 2021). As an example, Kovács et al. (2014) report higher valuations when services were offered by distinctive independent restaurants instead of standardized chain restaurants.

Service industries have their specific characteristics (Le et al. 2019). Unlike product industries with relatively predictable demand and considerable capacity flexibility, service industries face the issue of step-fixed physical capacity (at least over the short term), together with highly unpredictable time-variable demand patterns. This leads to e.g. a surplus seating capacity during low hours and insufficient seating capacity during peak hours (McGill and Van Ryzin 1999). Service providers balance fluctuating demand and revenues by employing revenue management (RM) tools, such as *dynamic pricing strategy* and the *control of the length of staying strategy* (Heo et al. 2013; Lee et al. 2021). However, customers’ attributions of scarcity may lead to different reactions to various RM tools, and further influence customer satisfaction. When scarcity is attributed to high demand, customers enhance price appreciation. As a result, they are more likely to accept the dynamic pricing strategy. However, when scarcity is caused by “control of the length of staying strategy”, customers feel disrespected (Lee et al. 2021), which consequently leads to low customer satisfaction (Guillet and Mohammed 2015; Lindenmeier and Tscheulin 2008).

### Socio-political research realm: resource scarcity in the water-energy-food security nexus

Scarcity in consumer markets is addressed in the socio-political research realm from the perspective of scarce material resources. Given the growing societal demand for physical resources, the whole world is facing an intractable scarcity issue (Steffen et al. 2015), that is, the recourse demand is going beyond the planetary boundaries, equity, and inclusivity (Rockström et al. 2009). Research works pursue a science-based paradigm to resolve the conflicts between human beings’ development needs and the planet’s resource scarcity. Specifically, the water-energy-food (WEF) security nexus emphasizes the *scarcity of water, energy, and food resources,* together with their intricate interrelationships (Hoff 2011). Researchers take a series of socio-political perspectives (such as political economy, environmental sustainability, and human development), to highlight their independencies and synergies of water, energy, and food resource sectors (Obersteiner et al. 2016; White et al. 2018). By doing so, scholars aim to optimize policy planning that negotiates the trade-off between global societal development and the ecosystem’s resilience maintenance. This section focuses on the natural resources scarcity dimension, and discusses each sector of the water-energy-food (WEF) security nexus, together with the identified interdependencies among the three sectors.

#### Research stream (F2) on “managing water scarcity”

Water is a scarce natural resource that is closely related to climate change and the future of human beings (Molden and Sakthivadivel 1999). Given the important role of water and its scarcity, researchers focus on water conservation strategies, so as to optimize water supply portfolios (Fraga et al. 2017). Well-known strategies such as financial incentives and political mandates fail to encourage water conservation in water-consumption scenarios (Zeff et al. 2020). Therefore, researchers aim to provide solutions for water management from the supply-side and demand-side.

Among different strategies for dealing with water scarcity, irrigation attracts significant attention from previous studies. Note that irrigation water is demanded not only in the agricultural sector (Pérez Blanco and Thaler 2014), but also in the urban sector (Hof and Blázquez-Salom 2015; Quesnel and Ajami 2019). Large landscape irrigation (with non-residential purposes) also accounts for a significant volume of water consumption (Morales and Heaney 2016). As a result, non-residential irrigation consumers become the research focus of recent publications. Quesnel and Ajami (2019) carried out a study in this regard. In this study, they investigate the dynamic water demand for non-residential irrigation consumers in the drought region; and find that policies help prompt long-term (e.g., yearly) water conservation behavior, but short-term (e.g., weekly) actions cannot be explained by political practices. Relatedly, Quesnel et al. (2019) compare the water use behavior of consumers with potable vs. recycled water. Results reveal that potable and recycled water consumers show the same demand pattern despite the different policies and pricing tactics.

#### Research stream (F4) on “footprint as a flow indicator of scarce resources”

Measuring resources consumption is the first step to guarantee appropriate utilization of the “planet’s assets”, so that the issue of resources scarcity can be alleviated (Qiang and Jian 2020). Taking water consumption as an example, scholars find that the final products only contain a limited fraction of water compared to the total volume of water used for the whole production process (namely, virtual water) (Allan 1997). With analogy to water consumption, the application of the “virtual” concept is expanded to various resources which are used for products and services production (White et al. 2018). To track both direct and indirect resources consumption, the notion of the footprint is introduced. Footprint assesses the total volume of a resource that is consumed during the production process of the goods and services consumed by various groups: individuals, households, companies, regions, or countries; within its spatial boundaries and embodied within its imports (Daniels et al. 2011). Using the input–output analysis, scholars calculate a variety of footprints, including water (Weinzettel and Pfister 2019), energy (Wang et al. 2020, 2019; Yu et al. 2018), and food (White et al. 2018) embodied at the international (Weinzettel and Pfister 2019), domestic (Wang et al. 2019), and (multi) regional levels (Wang et al. 2020; White et al. 2018). Based on the findings, decision-makers can formulate policies to rationally exploit, trade, and transport natural resources.

#### Research stream (F10) on “scarcity and food security"

Food security as a multidimensional concept relates to different types of scarcity. Food security includes three dimensions: availability (ability to provide an adequate supply of food), accessibility (ability to acquire enough food), and utilization (ability to absorb nutrients contained in the food that is eaten) (FAO, 1996), which are linked to supply-side food scarcity, scarcity of food access, and scarce nutrition support, respectively. Similarly, Beer (2013) categorizes the multiple dimensions of food security into two types. The first one is production-oriented food security, referring to the quantum of food available to people. While the second type is consumption-oriented security, representing the concerns about food access, health, and equity. Following the same line, risk management addresses the whole process from food production to food consumption.

Some studies specifically identified the risks of phosphorus scarcity to different stakeholders along the entire supply chain, ranging from environmental and management risks faced by food producers and traders to market-related risks encountered by food consumers (Cordell and Neset 2014; Cordell et al. 2015). Beer (2013) discusses the risk derived from uncertainty, scarcity, and value conflict in terms of both food production and food consumption. Other studies focus on only one sub-dimension of food security. For example, Wang et al. (2021a, b) explored the combined impact of risk perception and the access dimension of food security status on food consumption behavior during the Covid-19 pandemic. In sum, all these works address food-related scarcity issues to specific aspects along the supply chain of food production, distribution, and consumption.

### Other research realms

In previous sessions, we had a close look at the two main research realms of scarcity-related studies, namely, the consumer behavior research realm, and the socio-political research realm. Although the above-mentioned research realms can cover the majority of research topics, other dimensions of scarcity in consumer markets are also addressed from various other research realms, which can be summarized into three perspectives: economics, innovation, and operations management.

#### Competition effects of scarcity (F7)

The scarcity of managerial resources and capabilities leads to the competing interests of stakeholders in a business network (Greenley and Foxall 1996; Zhou et al. 2007). Studies talk about the conflicts between different stakeholders, and guide corporates to allocate scarce resources and managerial capabilities efficiently (Greenley and Foxall 1996). They emphasize the role of strategizing to address scarcity issues in competitive settings. Zhou et al. (2007) suggest that competitor-orientation strategies may improve performance in economically developing markets with scarce resources. Likewise, Crabbé et al. (2013) indicate that the economic crisis the companies are confronted with might hinder the investment in sustainable solutions, while the intention to meet customer demands drives companies to develop sustainable innovations of products and services.

#### Innovation effects of scarcity (F8)

Technological innovation offers various scarcity-related benefits, for example, fastening the production process, increasing the quality of the products, and lowering the manufacturing cost. Advances in technology have influenced and continue to play a role in solving scarcity issues in many industries (Shankar et al. 2021), such as the agriculture industry (Aubert et al. 2012), the retail industry (Kurnia et al. 2015), and the IT industry (Ghosh et al. 2019). When it comes to the retail industry, novel technology adoption has facilitated dramatic shifts in business models, whereby e.g. personnel shortages can be overcome by chatbots (Syed et al. 2020). Therefore, retail researchers focus especially on technology adoption in the subfield of e-commerce (Kurnia et al. 2015).

However, the scarcity of social resources may impede the adoption of innovative technologies even in cases of their objective superiority. For instance, the adoption of advanced agricultural technologies leads to higher productivity as well as less pollution, thus is identified as one of the most effective solutions to severe food shortages and environmental protection issues (Aubert et al. 2012). However, factors such as lack of information and/or scarcity of knowledge are recognized to cause the low adoption rate and the inefficiency of new technologies diffusion (Legesse et al. 2019). Given the heterogeneity in different industries, various factors are detected to hinder the new technology adoption. However, trust is the common factor that boosts technology acceptance in the industries we reviewed.

#### Optimization models of scarcity (F9)

Last but not last, Factor 9 focuses on studies in the field of operations research, aiming to provide solutions to a variety of scarcity issues in production processes. Findings from mathematical modeling can guide decision-makers to improve the operational performance of the entire supply chain.

In this topic, studies take the operational perspective to optimize the performance of supply chain processes, including supply, storage, distribution, and consumption. They focus on different scare scenarios, such as supply scarcity (Fleischmann et al. 2020), capacity scarcity (Koch 2017), financial resources scarcity (Totare and Pandit 2010), and the scarcity of demand–supply balance (i.e., oversupply or shortage) (Orjuela Castro et al. 2021). Using various model optimization methods, researchers aim to maximize economic profitability, improve consumer services and enhance the efficiency of the logistics network.

## Discussion and conclusion

Scarcity refers to situations where material or immaterial resources are not sufficient for needs satisfaction. In the last decades, scholars have investigated different facets of scarcity in different socio-economic areas, including (but not limited to) economics, politics, and social psychology (Fan et al. 2019). These studies in various disciplinary studies result in fruitful but rather fragmented work under the topic of scarcity (Shi et al. 2020). To connect disciplinary research areas and further facilitate knowledge exchange, the current paper provides a systematic review of the scarcity literature. Using exploratory factor analysis, we identified distinct research streams, which could be allotted into three prominent research realms.

The current paper captured various dimensions of scarcity in consumer markets, including the product/service scarcity in the consumer behavior research realm, the natural resources scarcity in the socio-political research realm, as well as the scarcity of managerial resources and social resources in other research realms (see Table 4 for details). Despite addressing various scarcity dimensions, studies in every research stream only focus on single scarcity dimensions, and are mainly located in single research categories.

**Table 4:** Matrix of research realm/stream, scarcity dimension, and research category

		Scarcity dimension					%articles assigned to WoS research category				
Research realm	Research stream	Product scarcity	Service scarcity	Natural resources scarcity	Scarcity of social resource	Scarcity of mangerial resource	Business & management	Economics	Engineering & green & sustainable science & technology	Hospitality, leisure, sport & tourisms	Environmental science and studies
Consumer behavior research realm: A two-sided view and scarcity	F1"Purchase-enhancing product scarcity cues"	√					72%	2%	2%	4%	0%
	F3"Dysfunctional effects of product scarcity on consumer behavior"	√					51%	12%	3%	4%	0%
	F5"Scarcity issues in the (broader) consumption context"	√	(√)				37%	2%	9%	7%	7%
	F6"Managing scarcity in the service industries"		√				36%	7%	3%	20%	1%
Socio- political research realm:	F2"Managing water scarcity"		(√)	√			3%	8%	22%	7%	15%
resource scarcity in the water-	F4"Footprint as a flow indicator of scarce resources"			√			7%	9%	20%	0%	17%
energy-food security nexus	F10"Scarcity and food security"			√			11%	7%	11%	0%	18%
	Competition effects of scarcity (F7)					√	22%	18%	11%	5%	9%
Other research realms	Innovation effects of scarcity (F8)				√		28%	3%	6%	6%	3%
	Optimization models of scarcity (F9)	(√)				√	34%	14%	6%	0%	2%

Remark: √ presents the majority of studies in the corresponding research stream;

(√) stands for the potential research directions with few or no related studies

As shown in Table 4, studies identified in the consumer behavior research realm are mostly located in *Business & Management*, *Economics*, and *Hospitality, Leisure, Sport & Tourism* WoS categories. Researchers paid particular attention to the effects of product/service scarcity on consumer responses. Two-sided effects of scarcity cues constitute the focus in this research realm. With a series of experiments, consumer behavior studies constructed a contingency framework for understanding the positive (negative) impact of demand-framed (supply-framed) scarcity on consumer responses. Accordingly, practical guidance was provided to marketers to motivate consumption behavior.

In the socio-political research realm, studies were centered on environmental sustainability. Researchers sought to resolve the planet’s natural resources scarcity from the angle of macro policies. Specifically, natural resource scarcity was investigated with a focus on the water-energy-food security nexus. Among all types of resources, water resources received the majority of attention from previous studies in this research realm. Moreover, as flow indicators of scarce resources, footprint-related methods were widely adopted to capture the visible and invisible resources consumption. Given the research subjects in this research realm, it is not difficult to imagine that most of the articles are assigned to *Engineering & Green & Sustainable Science & Technology* and *Environmental Sciences & Studies* categories. Finally, the mixed research realm (“other research realms”) covers scarcity studies with economic, innovative, and operations research focuses.

Despite the similarity with the consumer behavior realm in terms of WoS research categories, the mixed research realm has a different focus on scarcity dimensions and research objects. Studies in “other research realms” explored the scarcity of social/managerial resources within business activities. Thus, players (from producers to customers) along the industrial supply chain are the research emphases in this realm. By using mathematical modeling, researchers could strengthen the competitive advantages of the entire supply chain and its components.

**Fig. 3:**
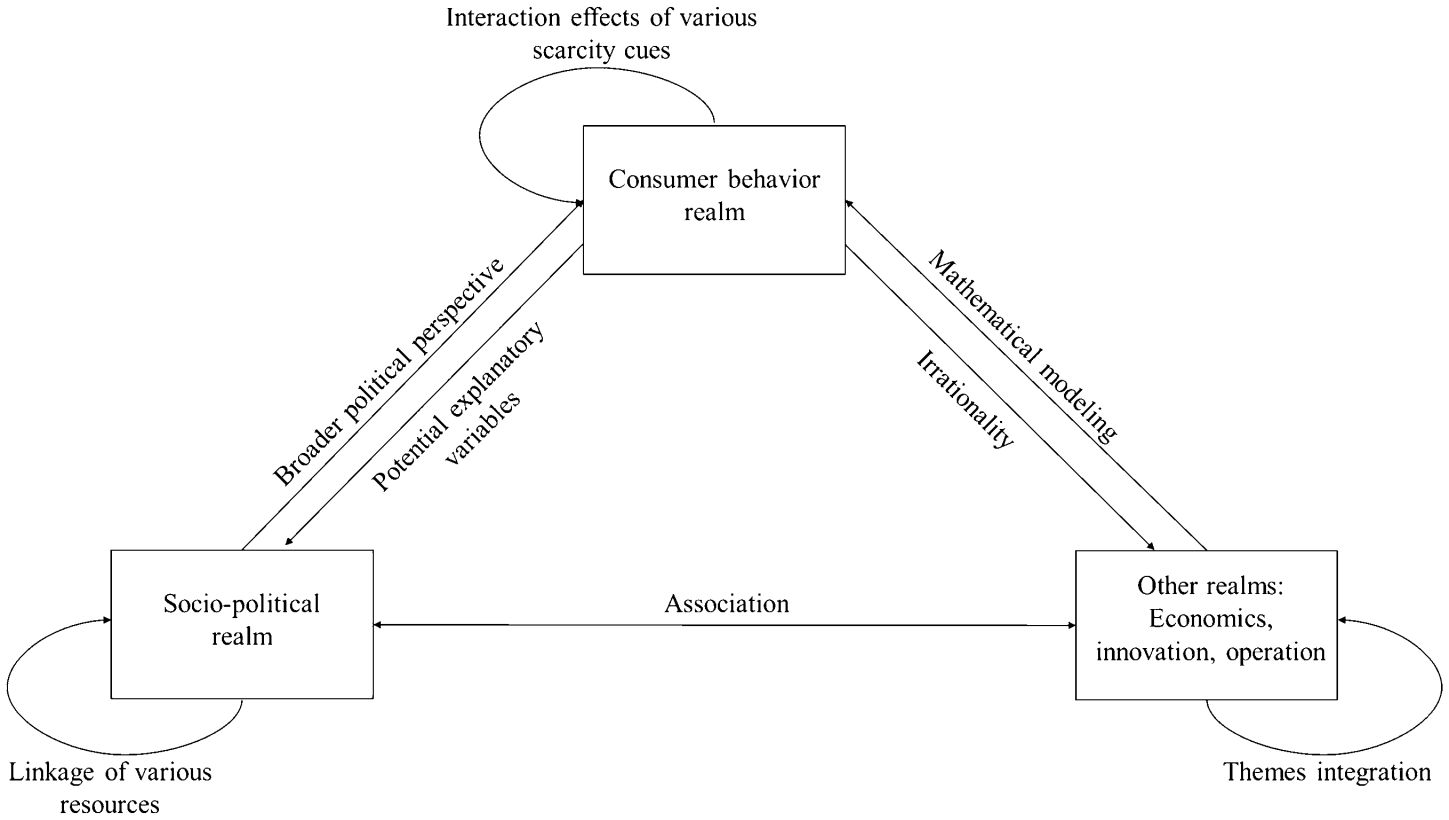
Future research inspirations for independent research realms and their convergence

Based on the identified research streams and the research realms they belong to, we further propose avenues for future research in each research realm. We also provide directions for interconnections of different research realms of scarcity studies. Figure 3 provides an overview of the future research inspirations at the research realm level and interdisciplinary level. These ideas will be outlined in the following, starting with research opportunities within research realms, followed by a discussion of transfer potential between the three research realms.

### Research opportunities within single research streams

Despite the surge of studies in consumer behavior research, some research gaps were still observed by our systematic review. First, most publications focus on one single type of scarcity cues, e.g., time pressure, financial constraints. Some studies have gone a step further by comparing product scarcity with different frames (e.g., supply-framed vs. demand-framed). Nevertheless, it might be interesting to investigate two or more types of scarcity cues at the same time, such as, to compare the consumer responses under the time pressure condition versus product scarcity (Song et al. 2021), to explore the interaction effect between two or more types of scarcity cues (e.g., limited-quantity & limited-time). The findings can contribute to research theories in this field, and further guide marketing practices. Second, although a series of interesting phenomena of scarcity have been reported in previous studies, they remain on the surface but fail to comprehensively reveal the corresponding psychological underpinnings (Peterson et al. 2020). Thus, it would largely enrich the literature if future studies could shed light on the cognitive and emotional mechanisms behind different phenomena.

In the socio-political research realm, water scarcity has drawn prominent attention from scholars. In obvious contrast to water, research in food (security) is "still in its infancy". The unbalanced focus, to some extent, contradicts the notion of the water-energy-food (WEF) security nexus, which emphasizes the integration of multiple resource sectors, and the optimization of the multi-goal models (Simpson and Jewitt 2019). Therefore, we encourage future studies to take a holistic perspective and consider the interdependencies of various resource sectors when conducting resource scarcity research.

In terms of “other research realms”, economic, innovative, and operational themes are mostly discussed independently. However, these concepts are not isolated. Instead, they connect and cause-and-effect each other (Filipescu et al. 2013; Zhou and Luo 2018). Given their reciprocal causality, the scarcity literature in “other research realms” could be enriched by more integration of research themes. For example, a previous economic constraint can hinder, postpone or even stop firms’ future innovation processes (Woschke et al. 2017). Lv and Qi (2019) found that the scarcity of innovative resources should be an important consideration in terms of the partner selection of the supply chain collaborative product innovation.

### Research opportunities by connecting research streams

Following the principles of scientific development, boundaries across various disciplinary studies on scarcity may become blurred over time as a result of knowledge exchange. The scarcity literature in different research realms can then benefit from each other’s findings, theories, and methods. By connecting seemingly unrelated research disciplines, researchers may take novel perspectives to ponder the unsolved research questions; studies might break away from inertial settings and analysis through knowledge integration; the practical implications in specific fields can be significantly enhanced. Under the topic of scarcity, studies that investigate different dimensions of scarcity focus on various research objects across distinct research categories (see Table 4). However, research objects, including consumers, firms, and society, compose a complete network in consumer markets. Firms can benefit from satisfying consumers’ needs, and consumers expect firms to integrate society's welfare into their corporate activities. In return, society ensures the sufficiency of materials and the financial resources for consumer-firm relationships (Peasley et al. 2021). Therefore, it is essential to explore the dynamic linkages among these research objects in various scarcity dimensions across different research categories.

Most of the studies in the consumer behavior research realm investigate how to present scarcity cues to prompt consumer purchase. The findings provide practical implications for marketers. Increasing purchase is often the only goal of such studies. However, according to previous studies on the side effects of scarcity, exposure to scarcity promotions may cause people’s aggressiveness and further increase crime (Kristofferson et al. 2017). Therefore, it’s harmful to focus on only marketers’ well-being. Consumer behavior studies should borrow a broader perspective from the socio-political research realm, and carefully consider the trade-off between positive and negative consequences brought by scarcity cues to different parties. Questions such as *whether scarcity cues should be manipulated by marketers*, and *to what extent they can manipulate scarcity promotions* should be answered (Hamilton et al. 2019). Such a socio-political view of researchers should guide policy makers to better regulate scarcity-related marketing strategies under the premise of ensuring consumer well-being and the interests of society (e.g., social stability) (Kristofferson et al. 2017).

Method-wise, studies in the consumer behavior research realm can benefit from a modeling approach widely used in “other research realms”. Most of the studies in the consumer behavior research realm don’t go beyond the traditional statistics. These methods are powerful to infer the basic relationships among variables, but cannot reveal the complex connections among variables, let alone provide accurate prediction results. Studies in “other research realms” have an advantage in this respect. Mathematical operations have shown their worth especially when variables have unpredictable or complicated interactions (Hannah et al. 2021). Thus, mathematical modeling can contribute to the studies in the consumer behavior research realm to untangle the intertwined relationships among variables. A good example would be the work of Luo et al. (2019). Following the advances of machine learning, the study adopts a causal forest algorithm to capture the complex heterogeneous effects between scarcity and price incentive. By doing so, the authors present a practical scheme to optimize the targeting strategies.

Studies in the socio-political research realm aim to manage the trade-off between societal development and resource scarcity by optimizing political planning. The majority of studies in this research realm analyzed objective secondary data from institutional databases, but ignored the self-reported data from customers, who are actually the resource consumption group. As a result, some potential customer-related explanatory variables (e.g., factors) are widely absent in these analyses (Villar-Navascués and Fragkou 2021). To bridge the gap, future studies could learn from the studies in the consumer behavior research realm, which take a perspective of consumers, and include consumer-related factors (e.g., psychological factors) to reveal the underlying behavioral mechanism. To be more specific, qualitative methods that capture subject data (e.g., interviews) should compensate for the current lack of measurement of customers’ subjective attitudes/perceptions. By combining subjective interview data and objective database data, researchers can better explain the reasons behind customers’ decision-making on resource consumption.

Environmental sustainability is the core of scarcity studies in the socio-political research realm. Researchers propose effective macro policies to regulate the sustainable management of natural resources. In other words, studies in the social-political research realm try to solve the scarcity problem at the society level, while most research in “other research realms” investigates the business organization at the enterprise level. These two research realms are quite irrelevant at the first glance, resulting in limited crossover research between them. However, the framework of the triple bottom line of sustainability reflects the connections between sustainability and business management (Dao et al. 2011). The framework states that the success of firms should be measured by their economic, social, and environmental achievements (Melville 2010). Thus, studies in the socio-political research realm and “other research realms” should notice the value of each other, and launch more discourses on the conjunctions between sustainability and business activities (Melville 2010). As an example, Melville (2010) explores how information systems can address scarcity by improving environmental sustainability through the belief-action-outcome path. As an extension of this work, Dao et al. (2011) combine the triple bottom line of sustainability and supply chain management, and argued about the importance of operations in entire supply chains in a sustainable manner. Accordingly, Bengtsson and Ågerfalk (2011) take a triple bottom perspective of sustainability to design sustainable logistical operations. Addressing scarcity along entire sustainable supply chains can address profit goals (cost/benefit maximization), people goals (stakeholder satisfaction), and planet (environmental impact) goals at the same time.

Last but not least, studies in “other research realms” tend to address scarcity issues by optimizing single aspects of business activities on the industry side. Optimal solutions for entire supply chains and each part are derived by mathematical calculations. However, people are the important components of corporations, and are the decision-makers for all business activities. As a result, managerial decisions always violate the assumption of rational behavior (Brouthers et al. 2008), and consequently, lead to imperfect or suboptimal decisions, especially under complex situations. Therefore, taking people’s irrationality into account when optimizing the interests of firms/supply chains brings more reality to business studies. Taking supply chain management (SCM) as an example, Wieland et al. (2016) argue SCM studies should not assume the decision-maker to be always objective and rational. Instead, SCM should develop models which can stand up to irrational agents and more complex decision-making (Sterman and Dogan 2015). Consumer behavior studies are quite advanced in this regard. A series of irrational behaviors (e.g., panic buying, unhealthy eating) have been measured and analyzed by studies in this research realm. In future research, “other research realms” studies could borrow the methods or theories regarding irrationality from the consumer behavior research realm to improve the robustness of their mathematical modeling when relaxing the rational choice assumption. One such example is the work from Sterman and Dogan (2015), which combines emotional factors (i.e., stressors arising from scarcity or poor supplier delivery performance) with operational modeling to explain customers’ irrational hoarding and phantom ordering.

## Limitations

Our paper is not without limitations. The current study focused on the socio-economics of scarcity. Yet in fact, scarcity was also investigated from an engineering perspective. We argue that it is already complicated enough to identify main research streams from the fuzzy studies in consumer markets, and propose the possible linkages among various research realms. Further studies may include a wider range of scarcity literature.

Although we carefully filtered out the irrelevant publications, it is possible that there are still some outliers in our dataset. Given the big amount of data, we argue it is impossible to individually check every publication by eyeballing. Nevertheless, due to the careful filtering and the low rate of outliers in such a large set of data, we believe that outliers can barely influence the robustness of our findings.

As a fast-emerging topic, scarcity has been attracting more and more attention from scholars. Every day new work springs up to contribute to scarcity research. The study’s findings are based on the most up‐to‐date literature available at that time. However, we do believe that some recently-published important work appeared after our dataset retrieval. Thus, we encourage future studies to review the most recent work to expand our research.

## Supplementary Information

Below is the link to the electronic supplementary material.

Supplementary file1 (DOCX 13 KB)

## Data Availability

On request.
