# Supplementary material for: Scarcity in today´s consumer markets: scoping the research landscape by author keywords
Source: Manag Rev Q. 2022 Sep 29:1–28. Online ahead of print. doi: 10.1007/s11301-022-00295-4 (PMC9520963; doi:10.1007/s11301-022-00295-4)
Supplement: Supplementary file 1 — Supplementary file1 (DOCX 13 KB) [file 11301_2022_295_MOESM1_ESM.docx]

Table S1. Author keywords in each research stream (factor) and research realm (complete table)

| # | Title | Keywords |
| --- | --- | --- |
| Consumer behavior research realm: A two-sided view and scarcity | | |
| 1 | Purchase-enhancing product scarcity cues | uniqueness, need desire, limited, luxury, conspicuous, self, scale, brand, cue, product, social, perceive, appeal, price, ad and persuasion, fashion |
| 3 | Dysfunctional effects of product scarcity on consumer behavior | choice, sale, promotion, psychology, response, effect, inequality, theory, consumer, marketing, preference, shopping, competition, economics, information, pricing, consequence, decision |
|  |  |  |
| 5 | Scarcity issues in the broader consumption context | green, intention, attitude, behavior, purchase, covid 19, plan, time, motivation, food, knowledge, consumer behavior, health |
| 6 | Managing scarcity in the service industries | satisfaction, quality, customer, hospitality, perception, online, social influence, retailing, value, service |
| Socio-political research realm: resource scarcity in the water-energy-food security nexus | | |
| 2 | Managing water scarcity | Water, use, demand, urban, conservation, irrigation, management, policy, region, climate change, water management, tourism, waste, climate, system, ecology, efficiency, cost, framework, willingness-to-pay |
| 4 | Footprint as a flow indicator of scarce resources | Footprint, virtual water, trade, carbon, China, energy, assessment, water scarcity, consumption, analysis, environment, agriculture, life, resource |
| 10 | Scarcity and food security | security, risk, impact, change |
| Other research realms | | |
| 7 | Competition effects of scarcity | corporate, innovation, orientation, performance, industry, sustainable, culture, strategy, market, development, economy |
| 8 | Innovation effects of scarcity | adoption, trust, internet, technology, communication, social media, network |
| 9 | Optimization models of scarcity | chain, supply, dynamic, design, model |
